# Struggling with one’s own parenting after an upbringing with substance abusing parents

**DOI:** 10.1080/17482631.2018.1435100

**Published:** 2018-02-26

**Authors:** Eva Tedgård, Maria Råstam, Ingegerd Wirtberg

**Affiliations:** ^a^ Department of Clinical Sciences Lund, Child and Adolescent Psychiatry, Lund University, Lund, Sweden; ^b^ Gillberg Neuropsychiatry Center, Institute of Neuroscience and Physiology University of Gothenburg, Göteborg, Sweden; ^c^ Departments of Psychology, Lund University, Lund, Sweden; ^d^ Offices for Healthcare “Sund”, Child and Adolescent Psychiatry, Infant and Toddler Unit, Malmö, Sweden

**Keywords:** Infant mental health, childhood experience, substance abuse, emotional abuse, parenting

## Abstract

**Aim**: To add to our knowledge concerning the key elements involved in the individual’s experience of growing up with substance abusing parents and the resulting challenges this involved for their own parenthood.

**Methods**: In-depth interviews were conducted with 19 parents who had participated in a mental health intervention programme. All had experienced substance abusing parents in their family of origin. Qualitative content analysis was used to analyse the data. They also completed a self-report questionnaire assessing their attachment style.

**Result**: Participants reported a high incidence of emotional abuse and neglect coupled with inadequate support from the community. Their own parental role was influenced by high parental stress and a majority had an insecure attachment style.

**Conclusions**: All participants had experienced a very difficult childhood which was reinforced by the fact that they received little support from society. Their childhood experience and the resulting challenges that this created in their own parenting role could negatively influence their own children’s ability to form a secure psychosocial development. It is therefore important to develop instruments that can help to identify children who were raised in misuse families in order to accommodate the transgenerational effects of growing up with substance abusing parents.

## Introduction

Children of alcohol- and drug-abusing parents belong to an especially vulnerable group and run a high risk of developing mental health problems. Parental substance abuse is often associated with family dysfunction, as the parenting skills and behaviours of adults with such problems are significantly impaired. Substance abusing parents can frequently be neglectful, abusive, unreliable, and emotionally unavailable for their children (Chassin, Rogosch, & Barrera, 1991; Velleman, Templeton, Reuber, Klein, & Moesgen, 2008). Children who are subjected to this kind of dysfunctional family situation, summarized in the concept of “Adverse Childhood Experiences” (ACE; Felitti et al., 1998), are at risk for developing problematic behaviours that affect both their own and their future children’s lives (Ivarsson, Saavedra, Granqvist, & Broberg, 2015; Kalmakis & Chandler, 2015; Lomanowska, Boivin, Hertzman, & Fleming, 2015). This study examines how individuals raised in such conditions reflect on their childhood and the kinds of difficulties that they have in their own role as a parent.

**Table 1.** Sociodemographic and psychosocial characteristics of the sample (n = 19).

	Mothers	Fathers
	n = 13	n = 6
Age of parent (years)	21–40	27–40
Primiparous	7	5
Age of child (months)	12–60	12–48
Married/cohabitating	9	4
Educational background		
Elementary <9 years	1	1
Grammar/secondary 10–12 years	7	4
College/university >12 years	5	1
Unemployed	1	1
Childhood in which both parents had substance abuse	4	2
Childhood in which mother had substance abuse	2	2
Childhood in which father had substance abuse	7	2
Parents separated during childhood	8	4

**Figure 1.**
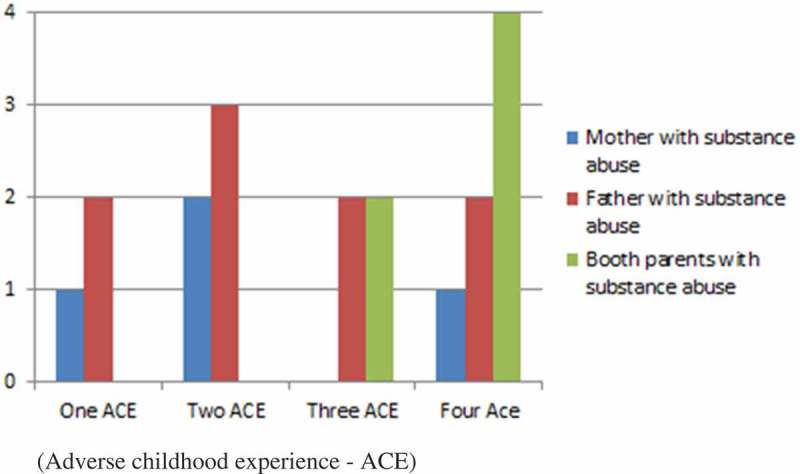
Prevalence of abuse and neglect during childhood (adverse childhood experience—ACE) in relation to which of the parents had substance abuse.

**Figure:**
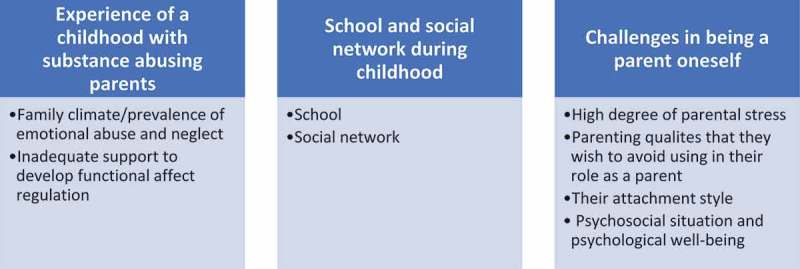

Adverse Childhood Experiences can take a number of forms and Felitti et al.’s definition includes *sexual abuse*, *emotional abuse* and *physical abuse*. Two additional aspects are *physical neglect* and *emotional neglect* (Felitti et al., 1998).

Growing up with substance abusing parents influences the child in many ways and studies have found links between parental substance abuse and the development of anxiety and affective disorders both in children (Hill et al., 2008) and in young people (Chen, Subramanian, Acevedo-Garcia, & Kawachi, 2005; Hill et al., 2008; Kelley, Pearson, Trinh, Klostermann, & Krakowski, 2011) as well as an increased risk of developing substance abuse (Buu et al., 2009; Johnson & Leff, 1999; Kendler, Ohlsson, Sundquist, & Sundquist, 2015; Yule, Wilens, Martelon, Simon, & Biederman, 2013).

An active substance abuse generates serious limits on the possibility to be a “good enough” parent. An infant needs sensitive and attentive caregivers who can interpret and respond to her different signals and who understand the intentions behind her communicative behaviour (Fonagy, Steele, Steele, Moran, & Higgitt, 1991; Slade, Grienenberger, Bernbach, Levy, & Locker, 2005; Sroufe, Egeland, Carlson, & Collins, 2005). In the first years the development of an attachment bond between the child and her parents is helped and strengthened by the infant’s affective communication (Schore & Newton, 2013). The quality of the attachment bond is dependent on the parent’s ability to encourage independence and curiosity in safe contexts, and provide safety and comfort in frightening situations (Cassidy, 2008). However, if the parent’s own behaviour is experienced as frightening this may well create an unmanageable situation for the small child, as the source of safety and support instead provokes fear (Main & Solomon, 1990; Solomon & George, 2011a; Van Ijzendoorn, Schuengel, & Bakermans-Kranenburg, 1999). According to attachment theory, inadequate caring of this sort seriously threatens not only the development of a secure attachment bond, but also the learning of many strategies that are important for competent interaction with others (Solomon & George, 2011b).

From birth onwards it is important that the child gets effective support to manage emotions (Bariola, Hughes, & Gullone, 2012). Children who have difficulties in this area are at risk for becoming either aggressive and hostile or isolated and socially anxious (Cumberland-Li, Eisenberg, Champion, Gershoff, & Fabes, 2003; Frick & Morris, 2004; Howe, 2005; Silk, Steinberg, & Morris, 2003). Children with a secure attachment system employ more adaptive emotion regulatory strategies than those who have an insecure attachment system (Mikulincer, Shaver, & Pereg, 2003). The emotional development of the child is to a great extent dependent on predictable patterns of caring, and she will at a very early age develop clear expectations concerning her relation with her parents (Beebe et al., 2010). There is a greater risk that the upbringing of children whose parents have an active substance abuse will be characterized by unpredictability, a high degree of stress and situations in which the parents are experienced by the child as frightening (Burnett, Jones, Bliwise, & Ross, 2006; Ross & Hill, 2004).

When these children grow up and become parents they may have difficulties in parenting. One of the most significant predictors of parenting behaviour is how parents were parented themselves (Anda et al., 2006; Conger, Belsky, & Capaldi, 2009; Main, Kaplan, & Cassidy, 1985; Meins, 1999; Slade, Grienenberger, Bernbach, Levy, & Locker, 2005). A number of studies have demonstrated that if a woman has a history of adverse childhood experiences, this will often be associated with difficulties in parenting (Moehler, Biringen, & Poustka, 2007; Sroufe et al., 2005; Steele et al., 2016; Tedgard & Rastam, 2016). According to the Avon Longitudinal Study of Parents and Children, adjustment problems in offspring were more chronic when mothers reported severe abuse during their own childhood. The effects were cumulative in that problems in offspring were greater when maternal exposure was to several types of abuse as compared to only one (Collishaw, Dunn, O’Connor, & Golding, 2007). For individuals, their personal attachment pattern is of significance in the development of their parental role. Studies show that insecure attachment styles are associated with a number of negative parental behaviours, such as lack of predictability (Coyl, Newland, & Freeman, 2010; Kilmann, Vendemia, Parnell, & Urbaniak, 2009) and lower levels of involvement (Coyl et al., 2010); (Feeney, 2002). In a review by Mikulincer, the conclusion is that securely attached individuals could use their feelings in a more constructive and positive manner when compared with individuals who display insecure attachment styles.

According to a large population-based study, about 17% of all children in Sweden live with parents with high risk levels of alcohol consumption (Hjern & Mahnica, 2013), and 3% of children grow up in families in which at least one of their parents has been diagnosed with substance abuse (Hjern, Arat, & Vinnerljung, 2014).

An investigation carried out by the department of Child and Youth Psychiatry in Malmö showed that approximately 30% of the mothers who sought help at the Infant Mental Health Unit had been brought up in families with substance abusing parents (Tedgard & Rastam, 2016). An earlier report had also shown that mothers who had grown up in families with substance abusing parents had more difficulties in relation to their infants, that their children demonstrated more symptoms and that it was more difficult to involve fathers in the treatment when compared to parents not brought up in such families (Tedgård, 2008).

### Aims of the study

The above findings served as a starting point for further investigating the experience of growing up with substance abusing parents and exploring in what ways it could be problematic. The aim of this study is to increase our knowledge and its effects on one’s own parenting. Are there common, key elements to be found in the descriptions the informants provided of their upbringing? How do they describe the challenges in their own parenthood? What are their main concerns in their role as a parent? This is essential information when trying to develop specific interventions for this vulnerable group of patients.

## Material and methods

### Subjects

The sample consisted of 19 parents who had participated in an infant mental health intervention programme in a specialized infant and toddler outpatient psychiatric clinic. The intervention programme has a combination of interaction treatment—where the parents are encouraged to become attentive to the child’s emotions and to their initiatives to make contact—and psychotherapy for the parent. From a total sample of 197 parents from the clinic who were screened by self-reports for sociodemographic and psychosocial data, a subsample of 53 parents reported that they had grown up in a family with substance abusing parents. From that sample, 29 parents who had concluded treatment were consecutively asked if they would participate in the study. Four parents declined, due to lack of time and/or a desire not to revive painful memories and two could not be reached by telephone. Twenty-three people agreed to participate and, of those, four did not show up for the interview, giving no explanation. The final sample (see Table 1) consisted of 19 informants (13 mothers and 6 fathers) whose children were between 1 and 5 years old at the time of the interview.

The subjects were invited by a letter that also contained information concerning the study. A week later they were contacted by phone and asked if they would like to participate. All participants gave their written consent and received two cinema tickets as a compensation for participating. None of the participants were partners with each other and none of the participants have received treatment from or had previous contact with the psychologist (ET) who carried out the interviews. In consideration of the possibility that informants might experience problems after the interviews, they were offered the chance to be referred for further counselling. One of the participants used that opportunity. Ethical approval was obtained from the Regional Ethics Committee in Lund, Sweden.

### Measures

The study had both a retrospective quality design, using in-depth interviews that focused on the subject’s upbringing, and a questionnaire with a cross-sectional design that measures current attachment style. The main methodological approach was qualitative—meaning that focus was on describing and understanding the life stories told at the interviews (Hyden, 1997; Launer, 2002).

#### In-depth interview

The in-depth interviews were conducted between May 2012 and June 2013. A semi-structured interview guide was used in order to access as many different aspects as possible while at the same time ensuring that everyone was asked the same key questions. The guide contained questions regarding experiences from childhood, including: family climate, relationship to parent(s), network and school. There were also questions concerning the present, and these included the experience of their own parenting. The informant was free to express herself and to associate in different directions to the open-ended questions, and efforts were made to get small narrative examples that illustrated the answers, in order to enhance understanding. The interviews lasted from 90 to 150 minutes. Each interview was tape-recorded after obtaining permission from the informant. Before the data analysis started the interviews were transcribed verbatim.

The questions asked focused to a large degree on the past and when asking people to recall the past there is usually no way of controlling “the facts”. The data collected are accepted as the memories and experiences that the women and men have of their childhood and upbringing. It is further assumed that their recollections of historical events, recalled in response to the retrospective questions of the interviewer, are influenced in some ways by the narratives they have in the present (Kvale & Brinkmann, 2009).

#### Attachment Style Questionnaire

The Attachment Style Questionnaire (ASQ; Feeney, Noller, & Hanrahan, 1994) has been translated into Swedish (Hakansson, 1996) and contains 40 items. The ASQ was designed to capture common themes in attachment theory such as trust, dependence and self-reliance in relationships in general (Feeney et al., 1994), as well as measuring basic personality factors such as the ability to have intimate and romantic relationships (Roisman et al., 2007).

The questions in ASQ can be analysed in two separate factors (secure and insecure attachment) but can also be analysed on the basis of a three-factor structure in line with Hazan’s and Shavers’s (1987) conceptualization of attachment (secure, anxious and avoidant). The ASQ answers ranged from 1 (totally disagree) to 6 (totally agree). Feeney et al. (1994) reported internal consistencies for the English version and found adequate Cronbach alphas for the subscales security (.83), avoidance (.83) and anxiety (.85). The test-retest reliability over a period of approximately 10 weeks was 0.74, 0.75 and 0.80 for the three subscales. The internal consistency for the three subscales in the Swedish version is in the same range as for the English original version (Hakansson, 1996).

### Data analysis

Qualitative Content Analysis (CA) was used to analyse the interview data (Graneheim & Lundman, 2004; Sandelowski, 2000). CA does not prioritize a search for deep implications, but rather aims to present a thick description of what was said in the interviews (Graneheim & Lundman, 2004).

The analysis itself is a stepwise process of categorization based on answers containing thoughts, feelings and memories as described in the texts. Keeping as faithfully as possible to the data is considered crucial, even if all analysis infers some degree of interpretation.

After carefully reading all the interviews several times, the text was divided into “meaning units”, which are those parts of the texts that relate to the aims of the study. All meaning units were then condensed while care was taken to preserve the original content. The condensed meaning units were then given codes. The codes were grouped into subcategories and thereafter into mutually exclusive categories, depending on similarities and differences in content. The emerging categories were then closely examined, discussed within the research team and reorganized until consensus was reached. Finally, we searched across categories to identify recurring regularities that could be expressed as a theme. The first author (ET) performed the analysis throughout the whole analytical scheme, and the last author (IW) participated in all steps after the coding process. The analytical process had a continual back and forth movement between the emerging categories and the original parts of the text in order to secure reliability and a comprehensive understanding of the material.

## Results

Our results reflect information gathered from two sources: the deep interviews in which the subjects were asked retrospective questions concerning their childhood experiences, and the results of the self-estimated questionnaire that examines the individual’s attachment style.

The material is categorized into three comprehensive domains. The first is “*Experience of a childhood with substance abusing parents*”; the second is “*School and social network under childhood*” and the third is “*Challenges in being a parent oneself*”.

### Experience of a childhood with substance abusing parents

Childhood experience is divided into two categories: (a) family climate, and the prevalence of abuse and neglect, and (b) inadequate support for the development of functional affect regulation.

#### Family climate and prevalence of abuse and neglect

Those aspects of the narratives that describe childhood upbringing reveal a family climate filled with fear, insecurity, aggression and a high degree of unpredictability: “*Mother was very considerate and loving and totally unreliable and erratic and very, very manipulative.*”

Except for one, all of the people interviewed had been the victims of emotional neglect, and 15 had suffered emotional abuse. More than half had been physically abused, and described shoving and blows, see Figure 1. None of the incidents resulted in reports to the social work authorities or visits to a health-care centre. One person described how her father had beaten her when she tried to stop him hitting her mother. Almost half described neglect in terms of lack of food, being left on their own or being forced to sit in pubs with drunken parents. Almost without exception they described their parents as being absent or non-attentive, manipulative, unpredictable and incapable of expressing love or affirmation to them.

More than half described how they had been afraid of their misusing mothers: “*Mother was like a ticking bomb. Once when I visited her she took me out to a restaurant and got really drunk, and then exploded for some reason*.” Almost half of the participants who had had a misusing father found the relationship so distressing in their teenage years, or later as young adults, that they avoided contact for periods, or even ended the relationship: “*Then dad started drinking, and there was always a really strange atmosphere, and you had to be careful. He got really strange and was often violent. Most of the time, I didn’t want anything to do with him.*” Several described the intense anger that the father would show if someone in the family named his misuse.

Subjects had different expectations concerning the nature of the care they wanted from their mothers and fathers. Those who had grown up with a misusing mother often expressed a desire to receive some form of emotional caring from her, a desire that was still felt even as an adult. The relationship to the misusing father, on the other hand, was described in terms of great emotional distance, and with scarcely any expectation that he would ever have significance for them. One person described his father as “*completely meaningless for our family. A friend said to me, ‘Your father sleeps in the bushes’. Really embarrassing. I mean, he existed, but he didn’t exist, it was mostly when he drank that he was sort of nice.*”

During their childhood, nine of the subjects experienced significant separations from one or both of their primary caregivers. Separations included placement in children’s homes or summer holiday homes, one parent who worked abroad for long periods of time, one who moved permanently abroad to live, and one parent who died unexpectedly.

A large majority of those interviewed described their parents’ relationship as being difficult and conflict-filled, often resulting in divorce or making it impossible for them to live together while the children grew up. Of those who grew up with parents who did live together, three stated very plainly that they wished that their parents had split up so that their upbringing could have been calmer (one lost his father early). Four of those interviewed said that the quarrels between their parents resulted in interventions from neighbours, from ambulance personal or from the police on a number of occasions.

#### Inadequate support for the development of functional affect regulation

The subjects described how as children they experienced great difficulties in understanding and dealing with their feelings, and that their parents were incapable of helping them.

Many told about difficulties in trying to deal with anger and aggression. Powerful and externalized explosions occurred that they could not control: “*When I was angry I broke doors or fought with my siblings … or hurt myself … as soon as things didn’t go the way I wanted it was a catastrophe, and glass, walls, everything just got in my way.*”

Other feelings, such as sadness or fear, were also difficult to deal with. A few of the subjects said that they were not allowed to express such feelings. One person said that happiness was the only emotion she was allowed to show. Several said that they had tried to shut off difficult feelings during their childhood, and continued to do so even now as adults: “*I got very good at shutting off my feelings. So, if there was something difficult, I could just push a button and feel nothing*.”

Many described feeling abandoned and generally feeling bad during their childhood. These feelings had persisted right up to when they started the intervention programme. Only three of the subjects said that they had been able to turn to their parents to seek comfort and support. A little over a third stated that anxiety and fear were the dominant feelings—which led three of the subjects to consider suicide or even to attempt to commit suicide: “*I built high walls, thick walls, so that when I was 18 or 19 I had no feelings at all. When I was a teenager, I thought about suicide at least twice a week*.”

That their parents fiercely denied the existence of their substance abuse was a further difficulty that resulted in the subjects being isolated with their own experiences, unable to talk with anyone about their home situation. This increased their feelings of fear and insecurity, and of being abandoned.

### School and social network during childhood

The results in this section are divided into two categories and describe the way in which the subjects experienced (a) their time at school and (b) their social network—which are the two arenas that, together with the family, contribute most to the individual’s socialization and idea of themselves.

#### Schooltime

Ten of the subjects said that they had had great difficulty in school. They described concentration and learning problems, frequent school changes, mobbing and truancy: “*I was bullied … had really poor examination results … couldn’t concentrate in school, was unruly, kept getting up to mischief. There was no peace and quiet for studying at home.*”

Those who experienced their school-time as good said that they got good results and that school was a place where they could get positive affirmation. Several described school as a refuge from the stressful situation at home.

Eight of the informants emphasized especially that it was thanks to teachers who took such a special interest in them that those teachers proved to be a decisive and positive influence in their development, and also made it possible for them to leave school with good grades:

*I wouldn’t have been able to get into high school I had such poor results, but I had a really nice teacher who helped me so that in the end I could get a place*.

*One important person was my junior school teacher. She took me under her wings. It was she who helped me to survive*.

#### Social network

All but two of the subjects reported that they had had friends of the same age during childhood, even if there were also periods when they were alone and bullied. Two described a childhood without friends.

While several of the subjects did not participate in any organized leisure time activities at all, others talked in positive terms about taking part in such activities—which also often included having important relationships that were outside the family: “*When I started to do sports, I was 13 or 14, and my trainer was really important for me. You get respect, and when I got married my trainer made a speech at the wedding, and we still keep in touch.*”

***Contact with relations and other adults.*** One important and recurring theme for many was the silence of the adults in their network concerning their home situation. A large majority of the informants said that they had never spoken to anyone about their home situation, and that they had absolutely no one with whom they could talk about their parent’s substance abuse. They said that they had had no support from either their relations or from the neighbours, and that not a single adult in their immediate circle acknowledged in any way that they were aware of their difficult situation. They strongly expressed the wish that there had been someone who had offered support to them in some way so that they could have felt less abandoned. Only two of those interviewed were brought to the notice of a social work agency.

### Challenges in being a parent oneself

The experienced challenges of being a parent are divided into four categories: (a) *high levels of stress*; (b) *parenting qualities that they wish to avoid using in their role as parents*; (c) *their attachment style*; and (d) *Psychosocial situation and psychological well-being*.

#### High levels of parental stress

The majority of the subjects described how they experienced being a parent as very demanding. Many of them said that they lacked constructive models for how a parent could be, and that they only knew that they wanted to be “*100% different*” from their own parents:

*I really dislike the way my parents were, and I don’t want to be like my mother at all*.

*I try to do just the opposite.*

*Together with my therapist I have written long lists that include all the things that I don’t want to do that my mother did*.

The high levels of parental stress took several different expressions and eight of the subjects said that they lived with strong feelings of anxiety and stress, and that they experienced threats and danger “*everywhere*”, and that this resulted in them being overprotective of their children. The feeling of anxiety could become dominant in the relationship to the child, and influence both interaction and intimacy. One of the people interviewed described feelings of such intense anxiety that it was difficult for her to be available for her child, whilst another said that everything revolved around her child to such a degree that she became extremely tired, and then stressed because felt that she was unable to cope:

*Being a parent is hard, I wish that I could feel happier. I am uncertain and worried that I won’t be able to learn them the things that I should … even in moments of happiness I feel a massive sadness, it’s really tough.*

I don’t trust anyone; I hear alarm bells in my head all the time … everything is dangerous.

***Difficult and challenging feelings.*** Another expression of the parental stress is recounted by those subjects who describe having difficulties connected to naming and managing their own negative feelings in relation to their children. They tended to have difficulties in achieving a balance between leading and following the child—becoming either too demanding or too compliant. One woman described how she sometimes wished that her child would just disappear. Another said that it was easy to start shouting and screaming at her child when she showed signs of unhappiness. Almost half of those interviewed described their own tempers as problematic, and that their ability to control their own irritation and anger was poor and this resulted in the child becoming the target for these feelings: “*They might be baking, for example, then it gets too much for me, and I get really angry and start shouting and screaming.*” Some described how they had difficulty in seeing their child become frustrated, and then wished that they could give the child everything that she wanted. Others said that that they did not want to force their children to do anything they did not want to do, and that they never wanted to make demands on them: “*I can’t deal with it when my son is sad. I am so soft, when he cries I just want to fix it. I would take down the moon for him. I want to do everything I can to make him happy. That is typical me.*”

***Difficulties in being separated from the child.*** Another difficult area described by about one-third of the subjects concerned separation: during even brief separations from their child, it felt as if their relationship—or even their own identity—was seriously threatened. These difficulties can be exemplified by quotations that are representative for six of the parents interviewed:

“*Aron was my comfort blanket, and I’ve been carried along on his back. What would I do without Aron? Who am I when I’m not Aron’s mother, when I’m just plain Anna?*”

One parent said, “*When I don’t think about her, I forget that she exists*”, which was a real obstacle for her to allow herself to be parted from her child. Another mother said: “*I have never been away from my children … no, no, I would die … I can hardly breathe if I just think about it.*”

***Guilt Feelings***. One final difficulty that reinforced the idea of being an inadequate parent for many was that they were gripped with guilt feelings if they were not continuously available and able to satisfy their child’s desires:

*I feel guilty when I am not together with my child, when I’m at work for example. Did I get children just to leave them with someone else?*

*I hardly ever feel that I am good enough, I am so preoccupied with everything that I do wrong, so that sometimes I can’t manage to think about all the good things.*

*I get a bad conscience because I am not at home with him more. I get a bad conscience for very, very much when it comes to my children.*

#### Parenting qualities that they wish to avoid using in their role as parents

In reply to the direct question concerning which of their parents’ characteristics they had found particularly difficult and that they did not want to be a part of their own repertoire of behaviour as parents, there were several answers. Many said that the emotional coldness, the silence and the lack of engagement that they had experienced from their misusing parents were qualities that they did not want to be present in their own parenting behaviour and thereby become a part of their own children’s developmental experience. Others talked about the aggressive atmosphere that was present in their family of origin, and of how frightening and difficult that was. The majority stated that they did not want their own children to have contact with the misusing parent.

#### Self-assessed attachment style according to ASQ

A majority of the subjects had an insecure attachment style. The most common pattern involved an ambivalent attitude towards relationships: on the one hand, they had difficulties in trusting others and being dependent upon them, whilst on the other hand they emphasized the importance of close relationships. The other pattern connected to insecure attachment style described relationships with other people as being distant, and insisted on the importance of being independent. This latter group also emphasized the importance of individual efforts and achievements, rather than relational qualities. A little more than one-third of the subjects had a secure attachment style, which meant that they answered that they had a trusting relationship both to themselves and to others, and that they were convinced that other people could be relied upon to help if it was needed.

#### Psychosocial situation and psychological well-being

Despite their difficult social and emotional upbringing, and the difficulties experienced in their parenting role, most of the subjects had created a practical and social life that worked to their satisfaction. At the time of the interviews, only two were unemployed, all had their own homes, just over half had finished high school, and six had graduated from university. Thirteen of the subjects lived together with the parent of their child. However, on the other hand, nine of the participants described how the mental health problems that they had experienced during childhood—mainly in the form of depression and anxiety—were still present at the time they began participating in the programme and influenced their parenting abilities in a negative manner. Four of the subjects reported that they had been in psychotherapy before entering the family programme.

## Discussion

The interviews revealed that common key elements in their upbringing were that they had grown up in the shadow of aggression and violence in their families, and that most of them had been the target of emotional and physical abuse. Emotional abuse has the most negative effect on a child’s development (Allen, 2011; Glaser, 2011; Iwaniec, Larkin, & Higgins, 2006), and is that form of abuse most likely to influence one’s own parenting in a variety of negative ways (Banyard, Williams, & Siegel, 2003; Muzik et al., 2013; Steele et al., 2016).

The majority of those interviewed reported that their parents had a very conflictual relationship, a factor that can be threatening to a child’s development. Children who witness destructive parental conflicts are at risk for long-lasting effects on both their mental health and on their ability to regulate their own stress—even when they have not been the direct victims of abuse (Cummings & Davies, 2010; Du Rocher Schudlich, White, Fleischhauer, & Fitzgerald, 2011; Gunnar & Fisher, 2006; Jessop & Turner-Cobb, 2008; Madigan, Plamondon, & Jenkins, 2016; Van Ijzendoorn et al., 1999).

The subjects revealed that they had not been able to talk with anyone about the difficult family situation that their parents’ substance abuse created; neither had they received any emotional support from anyone. More than half reported that they had experienced serious difficulties in school, but several also told about help they had got from engaged teachers, which meant that they were able to finish school with good examination results. When talking about their own parenting experience, they described high parental stress that took different expressions: they worried a lot, were very sensitive to possible dangers that could threaten their child, had problems being apart from their child, had difficulties in regulating their own feelings towards their child and often had strong feelings of guilt in relationship to their child.

It is important to note that despite growing up under difficult conditions with parents who had a clear substance abuse, the subjects received very little support from society. And this was a time—in the 1970s and 1980s—when social work agencies, school health-care and other health-care agencies in general were well developed in Sweden and were focused on the themes of parenting and offering support to children. Several factors might have played a role here. One is that emotional abuse is difficult to define and identify (Sneddon, Iwaniec, & Stewart, 2010), and even if members in the child’s network strongly suspect unfavourable and unsatisfactory conditions, it is still a large step to actually reporting such suspicions to the authorities. A recently published report from Sweden shows that while 7 out of 10 (70%) teachers had reason to suspect that certain pupils lived with misusing parents, only 2 out of 10 (20%) actually forwarded their suspicions to the relevant authorities (Systembolaget, 2017). In a parallel manner, 20% of physicians at local health care centres in Sweden admitted that they did not report children whom they suspected to be the victims of severe neglect (Talsma, Boström, & Östberg, 2015). In both cases, the professionals cited uncertainty in how to make a report, lack of support within their own organization and a low degree of trust for the social welfare authorities as reasons for not reporting their suspicions. Many teachers discussed their suspicions with student health-care staff or their colleagues without making a report to the proper authorities. Sixty per cent of the physicians reported that they did not even know if there was a protocol in their workplace to be followed in reporting cases of suspected neglect. Many of them also said they would like to have access to an expert with whom they could consult in such cases.

Another is that in families where the parents are misusers, there often occurs a form of role-reversal in which the child assumes responsibility for many parental tasks, including general care. When this happens, the child may become more sensitive to the needs of others than to their own, and may develop strategies that are perceived by observers simply as signs of competence—which in turn means that it can be difficult to be aware of the child’s own needs. This results in the child missing out on important aspects of their own developmental requirements (Macfie, Brumariu, & Lyons-Ruth, 2015). A third reason is that misusing parents often deny their misuse and react in in threatening and aggressive ways when it is alluded to. This can result in all of the family members cooperating in order to maintain the invisibility of “the elephant in the room” (Klostermann et al., 2011; Kroll, 2004). A fourth reason that can block the possibility of receiving help or support may be found in the deep sense of shame that is often felt within families where one or both of the parents are substance abusers. This shame means that family members often work hard to keep the misuse as a secret (Dearing, Stuewig, & Tangney, 2005). Nathanson describes how fear of abandonment and rejection are important factors in the development of shame (Nathanson, 1997). Children with misusing parents often experience being abandoned, and at the same time can feel ashamed that they are unable to get their parents to stop misusing (Rafferty & Hartley, 2006; Ryan, 1991). Similarly, both children and parents may also feel shame over the parent’s misuse, which contains the risk that they may be rejected by their own family, their own network and by society at large.

The factors described above may contribute in making the identification of substance misuse difficult, and thus may be an obstacle to obtaining possible help and support for the children who grow up in such families. There are self-rating scales; for example, Children of Alcoholics Screening Test (CAST) which is an established instrument that measures alcohol misuse in parents, that is available in both a long (J. W. Jones, 1983) and a short (Hodgins, Maticka-Tyndale, El-Guebaly, & West, 1993) version. Unfortunately, this test has not been validated for Sweden. However, there may be risks in using this type of instrument in contexts such as school health care: for example, it may be experienced as a violation of integrity, placing the child in an ethical dilemma when asked to reveal the nature of his parent’s substance abuse. Further, if a decision is made to use such instruments, there must also be sufficient resources in place so that any difficulties that may be discovered can be properly investigated and the possibility of relevant treatment made quickly available. Another way of offering support to this group of children is by making it available on different sites on the Internet (Andersson, Johnsson, Berglund, & Öjehagen, 2009; Strecher, 2007). One such site, well known amongst Swedish young people, is www.drugsmart.com. A net-based intervention for substance-using teenagers (WISEteens) has shown particularly positive effects for male adolescents (Arnaud et al., 2015).

### Challenges in one’s own parenting

As was noted earlier, the individuals who were interviewed were socially well adapted in most ways, despite having been raised under very difficult circumstances. Almost half, however, had or had experienced mental health problems, and for most of them it was when their first child arrived that they decided to seek help with these difficulties.

The change involved in becoming a parent for the first time is possibly one of the greatest that an individual makes in her life, and the requirements needed for an adult to become an adequate attachment figure for a child requires both the ability to be a “safe base” where the child can receive comfort and support as well as being able to encourage the child’s independence and investigative behaviour (Bowlby, 1982). A parent who has been the victim of serious neglect and abuse during her own childhood runs the risk that her own traumatic emotional experiences may be stimulated by the creation of the relationship with her own child (Fraiberg, Adelson, & Shapiro, 2003; George & Solomon, 2008). When this happens, it can be difficult for the child to obtain adequate contact. On top of this, the parent can feel a helplessness in relation to the child if she experiences that her own anxiety is easily stimulated, her feelings quickly run out of control and that she becomes stressed at the thought of being separated. When this happens, the child experiences difficulties in using the parent as a secure and safe attachment object (Hesse & Main, 2006). Instead, the child runs the risk of establishing an insecure or even disorganized attachment relationship with her parent (Solomon & George, 2011a). This risk increases if the parent has an insecure attachment herself (J. D. Jones, Cassidy, & Shaver, 2015), which a majority of the subjects interviewed her had. Parental insecure attachment style also correlates with high levels of parenting stress (Trillingsgaard, Elklit, Shevlin, & Maimburg, 2011; Vieira, Ávila, & Matos, 2012) .

The new orientation that a parent makes when a child is born is described by Daniel Stern in terms of four major themes. The first (“The life-growth theme”) is to ensure the survival of the child; the second (“The primary relatedness theme”) to form a deep emotional relationship to the child; the third (“The supporting matrix theme”) to establish a benign, protective network to help her achieve the first two themes; and the fourth (“The identity reorganization theme”) to develop an identity as a parent (Stern, 1995). Eight of those interviewed describe how they were overprotective of their child because they were worried that their ability to protect their child from the dangers that they saw everywhere was inadequate. This can be related to Stern’s first parenting theme which is to ensure the survival of the child. Fear of failure in this first area can have negative effects on how the other three areas develop. Further, if parents have difficulty in regulating their own anxiety, this can affect the child’s development in a negative way, and several studies describe a connection between overprotectiveness from the parent’s side and the growth of anxiety in the child (Bögels & Brechman-Toussaint, 2006; Macleod et al., 2008; Wood, McLeod, Sigman, Hwang, & Chu, 2003).

The second theme—to create a deep emotional bond with the child—can be difficult to achieve if the parent has difficulty in regulating her own emotions in relation to the child, as several of the interviewed subjects described they had. Children are dependent upon having positive emotional support from their parents in order to be able to understand and express their own feelings (Bariola et al., 2012) (Buckholdt, Parra, & Jobe-Shields, 2014; Zeman, Cassano, Perry-Parrish, & Stegall, 2006).

Even the third theme—to create a protective network around the child (which normally includes the family of origin)—was made more difficult when they had no trust in the ability of their misusing parent(s) to take secure care of a child, and therefore prevented contact between their child and her grandparent(s) as a protective measure.

The fourth theme—to develop a personal identity as a parent—becomes complicated when, as was the case with the present interview group, a majority (n = 14/19) explicitly stated that they themselves had only had negative role models, and that of the parental caring behaviour they had been the recipients of as a child there was very little that they wished to use in relation to their own child.

### Resilient children become parents

The individuals interviewed in this study are a group of parents who describe how they were raised under very difficult circumstances with different kinds of neglect and abuse, but who have to a large extent become socially well-adapted adults. However, when becoming parents, they experienced difficulties in child-rearing to such an extent that they sought infant psychiatric help. Moe (2002) discussed a phenomenon known as the “sleeper effect” which describes how while people who have been exposed to certain risk factors may seem to have adapted well, the emergence of new life situations can evoke severe problems. It is also possible that these subjects can be placed in the category defined by Emily Werner as “resilient children”. In her Kauai Longitudinal Study, Werner pointed out that about one-third of the children who had been raised under difficult circumstances succeeded quite well, and it was these that she called “resilient children” (Werner, 1995, 1996, 2005).

When Werner (1986) studied resilient children who had substance abusing parents, they found the following comon factors in their lives: they had a positive temparement that activated their primary caregiver; they had at least average intelligence; they possessed an ambition to create a better life than their parents; they had a good self-image; they had a belief in their own capacity; and they did not have a serious illness. In other studies, factors that influence the development of positive parenting skills for resilient children include the presence of a sensitive mother, the development of social competence, and the establishment of satisfactory love-relationships (Conger, Schofield, Neppl, & Merrick, 2013; Raby, Steele, Carlson, & Sroufe, 2015).

In the present study, most of the women and men interviewed stated that they had had a complicated relationship with their mother and did not count on the support of their father. Some also told of separations from their caregivers that they had experienced as negative. However, they also described supportive relationships with other adults such as teachers and other school staff, a neighbour, step-parents, youth club leaders and sports trainers that had been important for them, and slightly more than one-third had a secure attachment style. From the present study, we are able to note that almost all had good friendships during childhood, and that 13 of them lived in a stable relationship with the biological parent of their child at the time of the interview.

Many defined school as being important and a source of support, which probably means that school can be considered as an important “protective factor” for them. Involved staff are often important if children who come from challenged social backgrounds are to complete school successfully (Werner, 1996), and the significance of school as a partially compensating factor for such backgrounds is becoming more and more obvious in our postmodern society (Berlin, Vinnerljung, & Hjern, 2011; Vinnerljung, Lindblad, Hjern, Rasmussen, & Dalen, 2010; Werner, 1993). Nine of those interviewed told of valued leisure activities from which they received support, and these might also be characterized as protective factors that may have contributed to their development and adaption.

The subjects had the ability to reflect on the difficulties they experienced in their parent role, and this was coupled with the ability to seek out and accept help. The help that is offered should include strategies both for affect regulation and for lessening feelings of shame, as well as to create an atmosphere in which it is possible to talk openly about their parent’s substance abuse and the consequences that the abuse has had for themselves (Kroll, 2004). It is also important that treatment offers the possibility to develop a critical perspective concerning their parents’ parenting.

The ability to seek and receive help seem to be an especially important factor in terms of coping when children who have experienced abuse become parents (Egeland, Jacobvitz, & Sroufe, 1988; Egeland & Kreutzer, 1991), and is yet another factor—together with those listed above—which suggests that we may be able to consider the subjects of this group as “resilient children”.

## Limitations

Qualitative research has its own built-in limitations, and it is not possible to draw any specific causal conclusions between difficulties experienced in the family of origin and an individual’s own parental difficulties. However, the stories told offer a nuanced picture of individual experience. To interview people about their past means that we obtain subjective narratives of a childhood as it is remembered after as many as 10 or 20 years have passed. This introduces an uncertainty concerning the “objective” or “true” nature of the information received, but at the same time it is this information that the informants themselves use to try and make sense of their present situation (as we all do). When asking people to recall the past there is usually no way of controlling “the facts”. The data collected are accepted as the memories and experiences these women and men have of their childhood and upbringing. It is further assumed that their recollections of historical events, recalled in response to the retrospective questions of the interviewer, are influenced in some ways by the narratives they have in the present (Kvale & Brinkmann, 2009). There is an ongoing critique on how valid such data are (Widom, Raphael, & DuMont, 2004), while other studies support the use of retrospective data from childhood (Hardt, Vellaisamy, & Schoon, 2010).

Given the number of people who were approached and the actual numbers who participated in the study, there could be a selection bias. However, it might be considered reasonable to assume that at least some of the people who refused to participate did so because of difficult childhood experiences, and that their reason for not wanting to be interviewed was because they did not wish to waken and reflect on painful childhood memories.

It would have been interesting to focus on the subjects’ own parent role in greater detail. This possibility was limited for two principle reasons: first, that the informants had been parents for a relatively short time, and secondly, because of ethical considerations. Ethically, it was judged that the goal of the study did not justify the risk of causing extra anxiety and stress, even if participants were offered the possibility to get counselling after the interview.

A further limitation concerns the bias of the interviewer, who has worked for many years as a counsellor in the infant mental health intervention programme from which participants were selected. This means that, as a result of her experience, the interviewer had preconceptions concerning the nature and construction of the parenting role, something which may have influenced the choice and nature of follow-up questions in a context where treatment and research perspectives may influence each other.

## Conclusions

For the interview subjects, the experience of a difficult childhood with substance abusing parents was reinforced by the fact that they received little support from society. They described how in a childhood filled with parental substance abuse, neglect and in many cases violence, there was no reaction or initiative from anyone who was a part of their social network (neighbours, school, child-care authorities, etc.) to try to support and help them. The informants said that, as children, they experienced extreme difficulty in formulating and expressing a desire for help—often not even understanding that they were in actual need of help. They also told of feelings of shame and abandonment and of a longing that someone would signal that they saw the kind of situation that they were in. In many ways, they were invisible children. Unfortunately, contemporary research reveals that employees in both school health care and health care report their suspicions concerning neglected and abused children far too seldom (Systembolaget, 2017; Talsma et al., 2015).

In their role as parents, the participants generally were able to take care of their children’s basic needs of protection and practical care. The challenge they often faced as parents was in the psychosocial domain, and was characterized by difficult emotions that were hard to control, stress, and clear difficulties in the regulation of closeness and distance. These difficulties constitute a risk that their child will not be able to develop a secure bond with her parents, and instead have some form of attachment difficulty.

The above suggests the importance of training the different professions who are engaged in the domain of children’s health and welfare and of developing a relatively simple and easily administered method to facilitate consultation and cooperation between the education system, the health system and the social welfare system so that all can find it easier to fulfil their legal responsibilities and report their suspicions about children whose development and health may be in danger. For example, when family treatment methods are employed within the social services they should probably involve systematic investigation of previous or present misuse, violence and trauma in families of origin, in order to understand and accommodate the transgenerational effects that growing up in families where parents misuse may involve.
